# Hidden chamber discovery in the underground Hellenistic necropolis of Neapolis by muography

**DOI:** 10.1038/s41598-023-32626-0

**Published:** 2023-04-03

**Authors:** Valeri Tioukov, Kunihiro Morishima, Carlo Leggieri, Federico Capriuoli, Nobuko Kitagawa, Mitsuaki Kuno, Yuta Manabe, Akira Nishio, Andrey Alexandrov, Valerio Gentile, Antonio Iuliano, Giovanni De Lellis

**Affiliations:** 1 Istituto Nazionale di Fisica Nucleare, Sezione di Napoli, Naples, Italy; 2 Dipartimento di Fisica “E. Pancini”, Università degli Studi di Napoli “Federico II”, Naples, Italy; 3 Associazione Celanapoli, via Santa Maria Antesaecula 129, Naples, Italy; 4 ACAS3d Soluzioni Digitali, Via Siria 102, Grosseto, Italy; 5 Nagoya University, Furo-cho, Chikusa-ku, Aichi Nagoya, 464-8602 Japan

**Keywords:** Experimental particle physics, Archaeology, Scientific data

## Abstract

We report in this paper the muography of an archaeological site located in the highly populated “Sanità” district in the center of Naples, ten meters below the current street level. Several detectors capable of detecting muons - high energy charged particles produced by cosmic rays in the upper layers of atmosphere - were installed underground at the depth of 18 m, to measure the muon flux over several weeks. By measuring the differential flux with our detectors in a wide angular range, we have produced a radiographic image of the upper layers. Despite the architectural complexity of the site, we have clearly observed the known structures as well as a few unknown ones. One of the observed new structures is compatible with the existence of a hidden, currently inaccessible, burial chamber.

## Introduction

**Figure 1:**
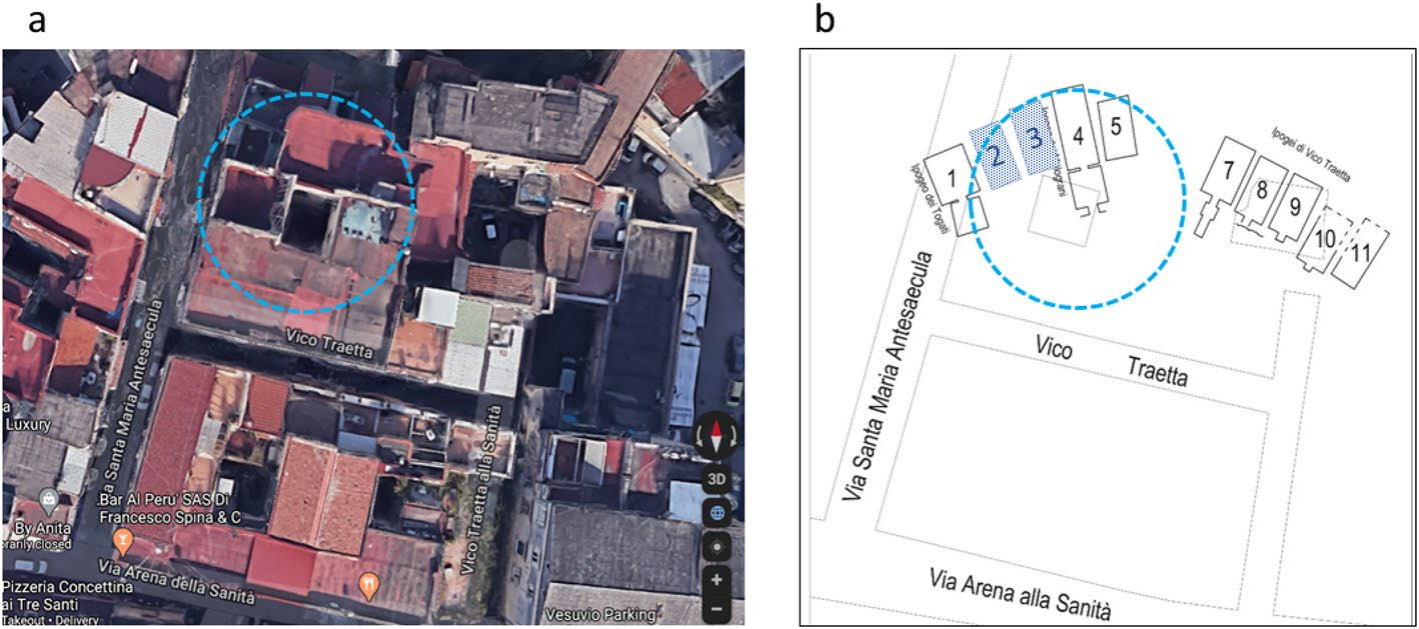
(**a**) Foreground top view of the studied site as seen with Google Maps ^25^. The dashed circle corresponds to the region explored in this paper by the muography technique with two detectors. (**b**) Schematic drawing of the underground level at the depth of 10 m with Greek funeral chambers numbered from 1 to 11. Scale and orientation in (**a**) and (**b**) are the same. Chambers 2 and 3 are unknown and their existence was hinted by the integrated site topology reconstructed with 3D surveys. (Satellite imagery: Google $\copyright$2022 Imagery date 7/29/2020).

**Figure 2:**
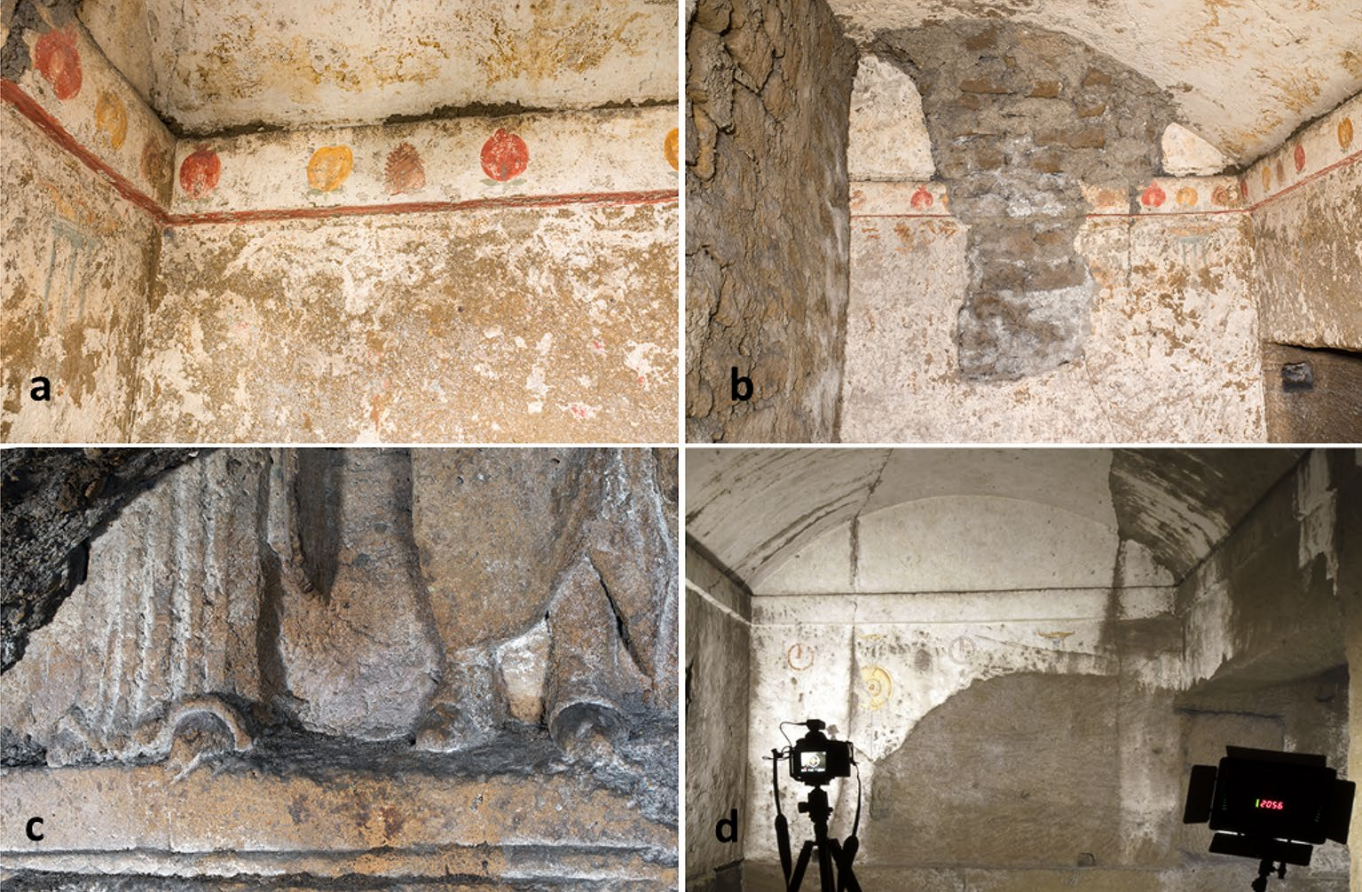
(**a**) Fragments of Greek burial chambers and (**b**) Ipogeo dei Melograni decorated with fruits frescoes along the walls (**c**) Togati - fragment of a high relief with a funeral farewell scene (**d**) chamber 8 in Fig. 11 with remains of frescoes on the North wall described by Neapolitan archaeologist Michele Ruggiero in 1888.

Remains of the ancient Neapolis with its buildings, streets, aqueducts and necropolis made by the Greeks starting from the second half of the first millennium BC are interred approximately ten meters below the current street level of the city of Naples. A little part of this archaeological treasure is accessible, thanks to the underground structures like water cisterns made since the sixteenth century or bomb shelters build during the Second World War which accidentally cross ancient cultural layers. Systematic archaeological excavations are not always possible in Naples, mainly because of safety concerns for buildings and streets in its highly populated districts. The *Ipogei dei Togati* and the *Ipogei dei Melograni*, denoted in Fig. 1 as chambers 1 and 4, respectively, are two known Greek burial chambers found below the Sanità district of Naples. The tombs are part of the ancient necropolis developed in this area in the VI–III century BC. Beautiful frescoes and alto-relievos were found in these ancient monuments created by wealthy Hellenistic families as shown in Fig. 2. In this area, covered later on by a thick alluvial layer hiding all memories of ancient preexistence, a fast-growing urbanization has been developing since the XVI century. The new constructions, while intersecting the ancient monuments, have often incorporated or partially destroyed them. Despite the profound transformations of this area over time, recent studies allowed to figure out the original morphology of the ancient necropolis landscape with burial chambers, developed along the road originating from the North Gate of Neapolis. The study of the integrated site topology with 3D surveys led to the hypothesis of a possible presence of additional burial hypogea as part of the Hellenistic necropolis, as shown in Fig. 1. In order to investigate this hypothesis, in this paper we have explored the site with the muon radiography technique. This modern technique consists of measuring the differential flux of muons, elementary particles naturally produced in the upper layers of the Earth atmosphere. The angular and momentum spectrum of muons, their flux, as well as their propagation length through different materials are well known. Therefore, this perpetual muon rain on the Earth surface can be used for the radiography, hence the so-called muography, of massive targets such as volcanoes ^1–4^, underground cavities ^5–7^ and Egyptian Pyramids ^8,9^. Due to its non-invasive nature, this technique is particularly suitable for urban environments where the application of active inspection methods such as seismic waves or boreholing is not conceivable.

**Figure 3:**
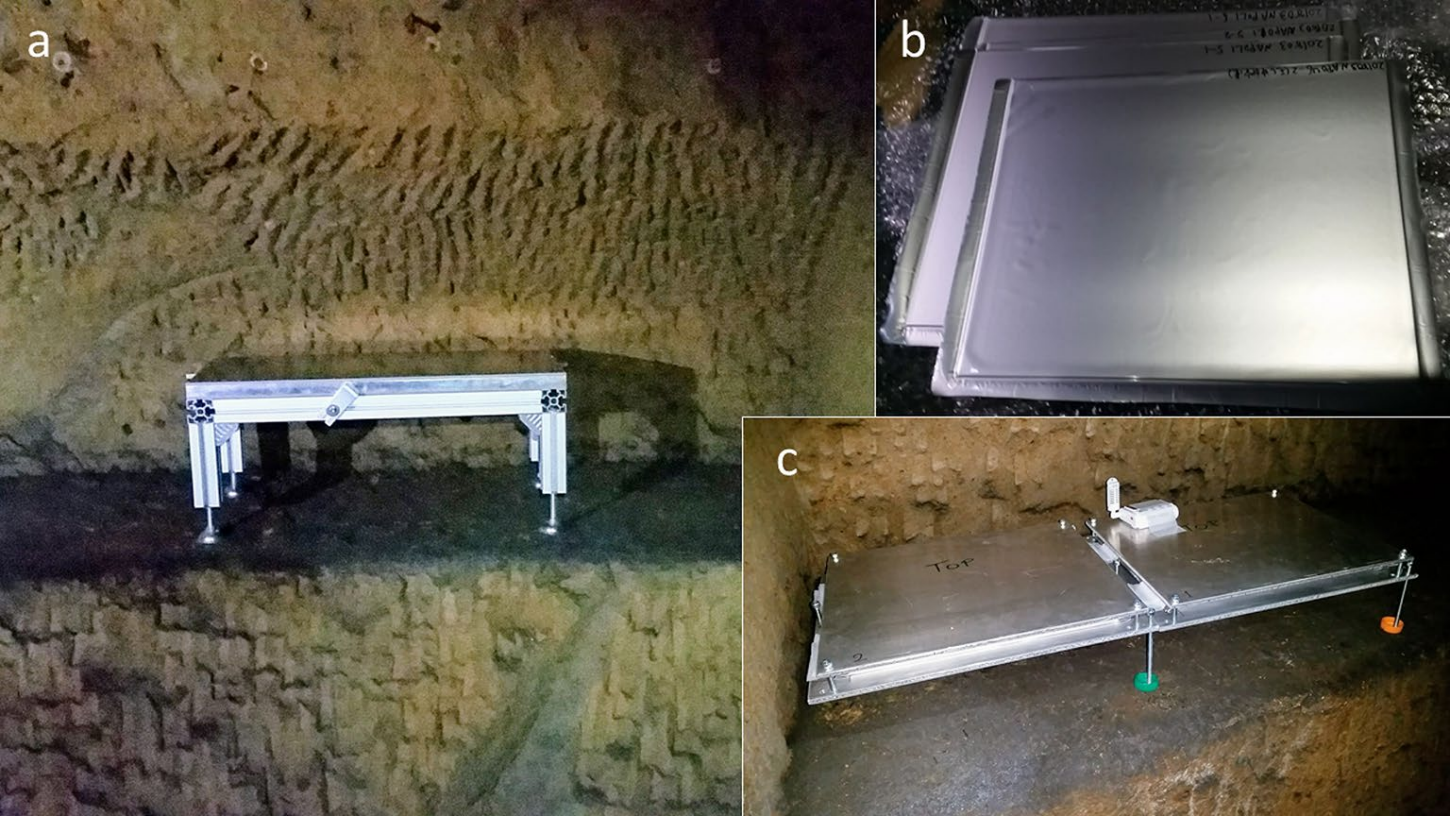
(**a**) Emulsion detector VP2 while taking data installed underground in the U15 “Chianca” room at 18 m depth. (**b**) Emulsion plates sealed inside protective envelopes.(**c**) Emulsion detector VP1 taking data in parallel in a different position inside the same cellar. The total sensitive area of each detector is 1500$~cm^2$.

**Figure 4:**
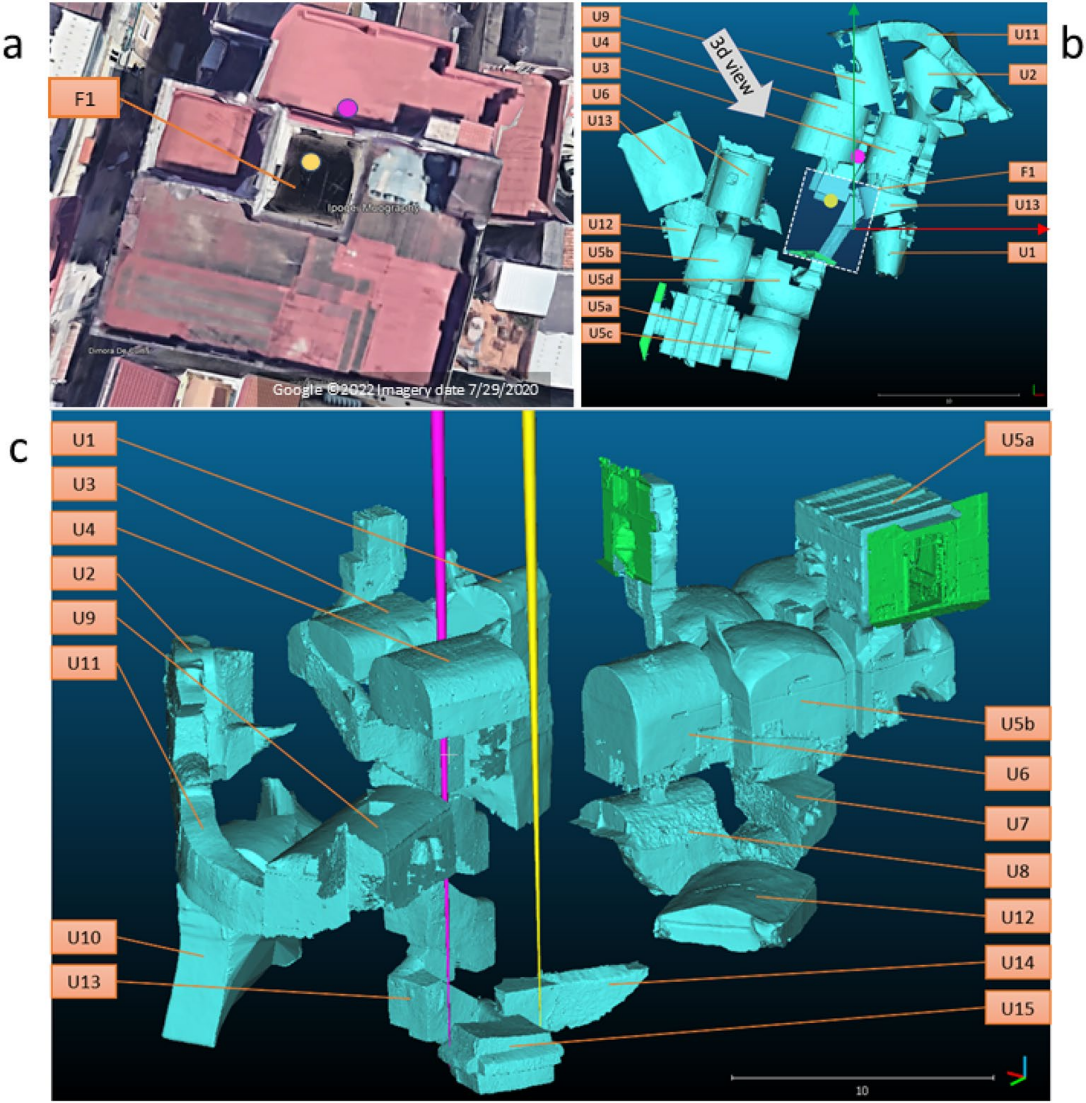
(**a**) Top view of the site as seen with Google Maps. (**b**) Top view of the underground part of the site as implemented in our model: the white dashed line indicates the position of a courtyard between buildings. The model reference system is indicated by red (x) and green (y) axis crossed at the origin corresponded to the street level in z. (**c**) 3D view of the underground part of the model taken from the direction indicated by grey arrow on (**b**). All known and accessible structures defined in Table 1 are shown here. Yellow and purple cones indicate the location and orientation of the two detectors VP1 and VP2, respectively. (Satellite imagery: Google $\copyright$2022 Imagery date 7/29/2020).

## Results

### Experimental setup

We have used a detector based on the nuclear emulsion technology ^10^, featuring the highest spatial resolution in measuring ionizing particle tracks. Nuclear emulsion is composed of tiny silver bromide crystals immersed in a gelatin binder. The crystals act as sensors that are activated by the ionization loss of a passing-through charged particle. The activated state of the crystals is preserved until the emulsion film is chemically developed. Thus, a particle track is recorded, first as a sequence of activated crystals, which later, after the development, becomes a sequence of silver grains. The formed tracks are visible at fully automated optical microscopes where their position and direction are measured.

Emulsion detectors are simple, extremely compact and, unlike electronic detectors, they don’t require any power supply ^10^ or gas feeding system. This makes them particularly suitable in a harsh environment such as in underground installations or on volcanoes. We used two detector modules shown in Fig. 3 and assembled with the same structure, consisting of a pile of four films, $25 \times 30~cm^2$ wide and about 300 $\mu$m thick. Each emulsion film was sealed inside an envelope for light and humidity tightness. The film piles were placed between two flat aluminum plates, acting both as protection layers and as the mechanical frame of the detector. A thin soft rubber layer was added between the top plate and the emulsion film to distribute uniformly the pressure and protect sensitive layers against any mechanical stress.

**Figure 5:**
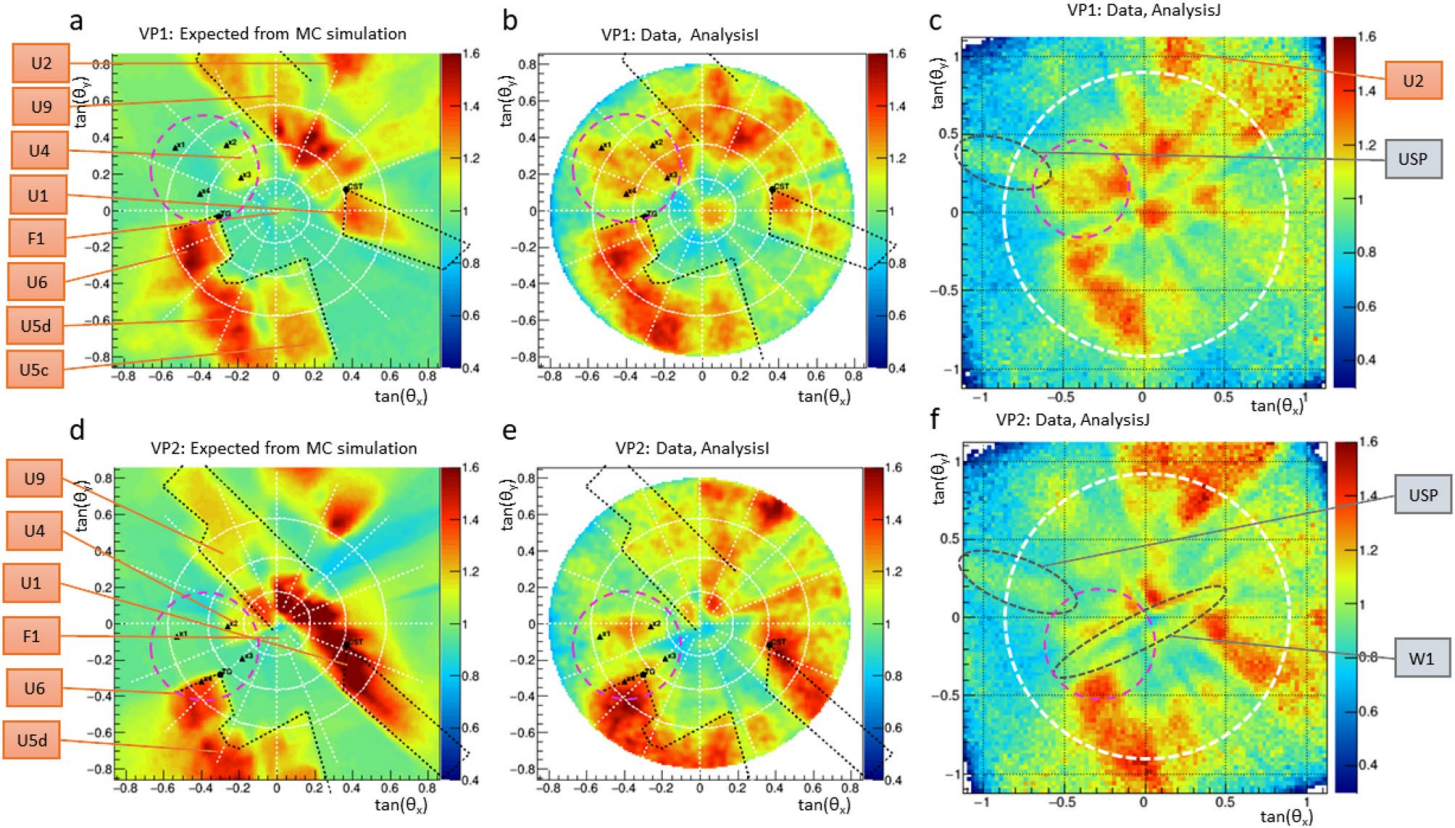
Muography images reporting the muon flux simulated using the 3D model (**a**,**d**) and measured (**b**,**c**,**e**),**f**) all normalized to the one obtained by MC at the detector depth in the assumption of a bulk rock. Color scale represents the relative flux excess with respect to the one without cavities. (**a**–**c**) show the expected and measured *AnalysisI* and *AnalysisJ*, respectively, for the VP1 while (**d**–**f**) show the same quantities for the VP2. The white dashed circle in the (**c**, **f**) plots shows the angular acceptance of the *AnalysisI*.

**Figure 6:**
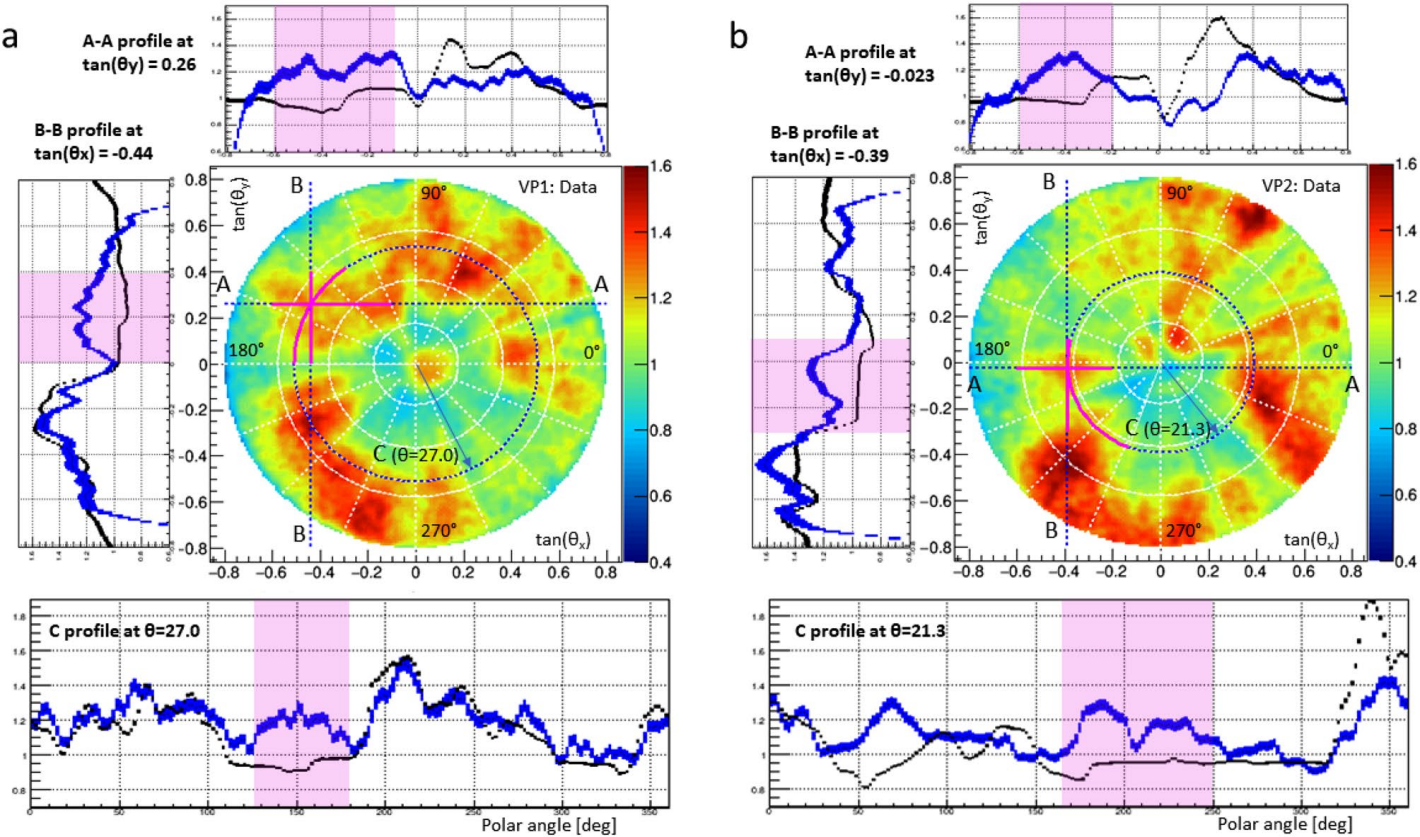
(**a**) The plot in the center is the bi-dimensional muography plot, same as in Fig. 5b. (**b**) The plot in the center is the same as in Fig. 5e. Both plots are accompanied with 3 profiles corresponding to the section lines A-A. B-B and the circumference C. Solid pink segments indicate the anomaly span. On the profile plots these regions are highlighted, the blue line stay for data, black for simulation (shown on Figs 5a and  5d), the vertical error bars represent the statistical uncertainties defined by the bins population with muons. The angles are given in degrees (0–360), the polar one follows east-counterclockwise convention.

**Figure 7:**
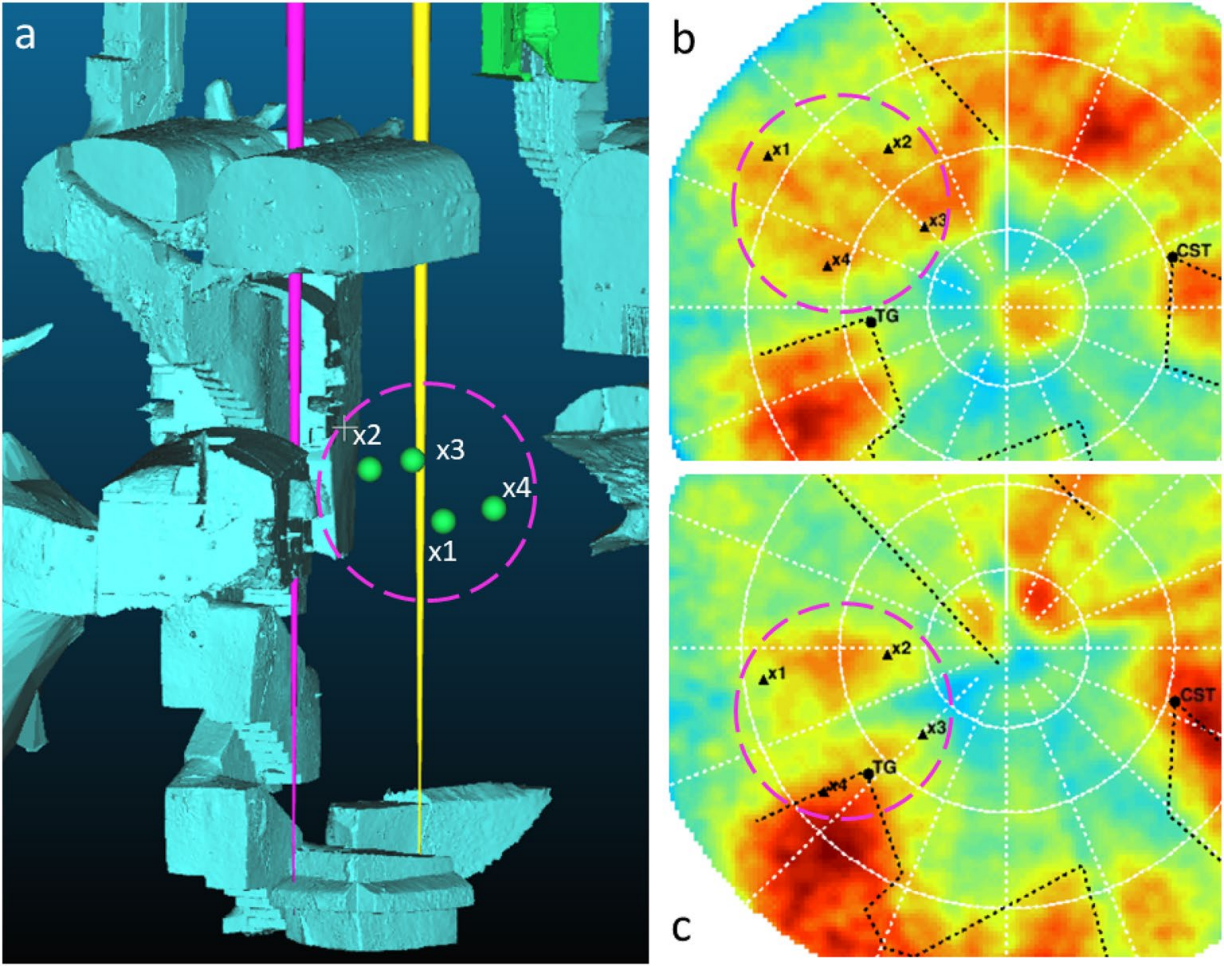
(**a**) 3D view of the site with the four inferred reference points $x_1$ to $x_4$ reported as green spheres. (**b**,**c**) are the zoomed view of the region of interest as seen from the two detectors. Black triangles present the projections on the muography image of the 3D points $x_1$ to $x_4$.

The modules were kept horizontally for 28 days in the period March 10 to April 7, 2018 inside the so-called “Chianca” underground room, a cellar used in XIX century for ham aging, denoted as U15 in this work, at a depth of about 18 m below the street level. At the end of the exposure the piles were disassembled and the films were kept in a different order during their transportation. Emulsion films register all the charged particles crossing the sensitive layers until they are developed. In a single emulsion plate tracks registered during the exposure period cannot be distinguished from those integrated elsewhere. To solve this ambiguity, we typically use a stack of two or more emulsion films placed in a given order and the pattern matching procedure to identify those tracks integrated during the exposure. This procedure shows a purity higher than 95%.

All emulsions were developed in Naples next day after the detector extraction.

Normally two consecutive plates are sufficient for unambiguous reconstruction. In this experiment stacks of four films were divided in two doublets. Each doublet was independently analysed in one of the two scanning laboratories equipped with high performance scanning systems: Naples (Italy) and Nagoya (Japan). Both the scanning and analysis chains applied in two laboratories were independent. The final results are fully compatible, confirming the high quality of both processing. To distinguish between these two analysis chains, hereafter we refer to *AnalysisI* and *AnalysisJ* for Naples and Nagoya, respectively.

### Digital model of the site

As it is the case for medical X-ray radiography where the recognition of anomalies relies on the precise knowledge of the body structure, also for the muon radiography the recognition of hidden hollow structures requires a 3D model of the known structures. This is particularly important in the investigation of such a complex and multi-layer environment of the underground structure of Naples. There are stairs and cisterns spanned from the depth of 20 m up to the surface level; the historical layer, target of this investigation, is at the depth of $8\div 12$ m; concrete basements of the buildings are rising up from the depth of about 6 m; cellars, underground sewer and connections, street level structures and the buildings themselves have to be described in the model.

**Table 1:** List of all accessible underground structures.

ID	Title	Level
F1	Foreground: external courtyard between the buildings	0
U1	Cistern in front of the Melograni chamber	−1,−2
U2	Cellar at the end of a modern ramp	−1
U3	Cellar above the Melograni	−1
U4	Cellar above the detector room	−1
U5	Celanapoli cellar	−1
U6	Celanapoli cellar	−1
U7	Togati hypogeum lobby	−2
U8	Volume below U6	−2
U9	**hypogeum n. 4 - Melograni burial chamber**	−3
U10	**hypogeum n. 5 with the floor collapsed during excavations**	−3
U11	Modern ramp	−3
U12	**hypogeum n. 1 - Togati burial chamber**	−3
U13	Stairs vertical volume	−4
U14	Impasse corridor	−4
U15	Chianca where detectors were located	−4

Levels are defined as follows: 0 for the street level on the surface, −1 for cellars at the depth of $2\div 4$ m, −2 at the depth of $5\div 6$ m, −3 for the Hellenistic necropolis at the depth of $7\div 10$ m; −4 for the lowest structures at the depth of $14\div 18$ m. Bold text emphasizes the structures belonging to the Hellenistic historical layer.

Muography produces overlaid images of all the layers above the detector position, making the detection of unknown structures rather challenging. A dedicated laser scanning campaign was carried out by the ACAS3d company in this work, as part of the Italian cultural heritage program for digitizing historical and archaeological monuments. The survey was extended to all ancillary underground spaces such that a precise spatial model containing all the accessible underground structures was produced, as shown in Fig. 4. This figure shows both foreground (a), and underground (b) and (c) parts of the site. Moreover, narrow cones with their vertices in the center of the detector and their axis along the vertical direction are drawn to indicate the two detectors and their prospective views: hereafter we identify the detector positions (View Points) as VP1 and VP2, corresponding to yellow and purple cones respectively. In order to easy the navigation across the site and in the following plots, we have assigned in Table 1 a unique identifier to all the known underground locations. This 3D model of the site was introduced in a Geant4 based simulation to describe the particle propagation through matter and produce the expected muon flux at the detector. This procedure is described in detail in the “Methods” Section.

### Data compared with the expectation from known structures

The plots in Fig. 5 show the expected (a, d) and measured (b,c,e,f) muon flux, all normalised to the flux obtained by MC at the detector depth in the assumption of a uniform bulky rock with no cavities, as a function of the projected slopes of the muon tracks, i.e. $\tan {\theta _x} = {\Delta x}/{\Delta z}$ and $\tan {\theta _y} = {\Delta y}/{\Delta z}$ where z is vertical (Zenith) axis. The expected flux at the location of our detectors, at the depth of 18 m, is obtained by including in the simulation the detailed 3D model reported in the previous Section. Top plots in Fig. 5 are for VP1, bottom plots for VP2.

These plots provide a view of the site similar to that one could take with a camera installed at the same location of our detectors, if rocks were partially opaque to the visible light, the opacity being proportional to the rock density. The color scale shows the amplitude of the flux ratio: the green color (ratio approximately equal to 1) indicates a muon flux compatible with the hypothesis of no-cavity in the given angular bin while a yellow-red color shows an excess due to cavities intercepted by the particles coming from those directions. The images of the most relevant underground structures are clearly visible here and are marked with corresponding identifiers. Black dashed contours are projections of the 3D lines indicating the shapes of some structures: U5, U6, U9 (Melograni) and U1 (cistern). Comparing left and right plots, one can clearly see the very close similarities of most of the structure shapes such as U5, U6, and U1. Not all regions show a perfect correspondence between data and expectations, due to some missing details such as the density variation in the underground layers and the wastewater network under the street. The most important missing details are load-bearing walls and the concrete foundations of buildings. These details contribute to the suppression of the muon flux especially in the low angle region, within 10 degrees from the vertical direction. In particular, the VP2 happens to be below the load-bearing wall of the building, and this is clearly visible in the plots (e, f) where the hardly suppressed region in the center is highlighted by a dashed grey ellipse “W1”.

**Table 2:** Positions of the detectors and coordinates of reference points in the reference system used in the paper (in meters).

ID	X	Y	Z
VP1	−1.66	1.76	−18.2
VP2	0.26	4.92	−18.2
x1	−4.19	7.18	−9.0
x2	−2.09	6.36	−8.0
x3	−2.44	4.43	−8.0
x4	−4.48	4.61	−9.0

Apart from these differences attributed to some details missing in the 3D model, the most interesting anomaly detected in this analysis is the one inside the pink dashed circle drawn on all 6 plots between U6 and U4. There is no known structure expected to produce an increase in the muon rate in the region on the left of U4 as it is visible in the plots (a and d). Nevertheless, a clear excess in that region is found in the data, and reported in the plots (b, c, e and f). The reference points $x_1$, $x_2$, $x_3$, $x_4$ on (a, b, d, e) indicate the boundaries of this region, as projections of 3D points on both VP1 and VP2 muographies. These 3D points are denoted as small green spheres in the 3D view of the site reported in the a) picture of Fig. 7. Plots (b) and (c) in the same Figure show a zoomed view of the anomaly region for both muographies. The observed excess is compatible with a cavity $2\div 3$ m height, located in the volume delimited by the $x_1-x_4$ points. The coordinates of reference points and the detector positions in the 3D-model reference system aligned with data are summarized in Table 2. The mean depth of the anomaly is of 8.5 m below the surface and the distance between points is $2\div 3.5$ m, which characterize the cavity dimension. The projection of the anomaly to the muography shown in plot b) is partially superimposed to U4 while in plot c) there is a partial overlap with U6 located on the upper levels. In the plot c) of Fig. 7, it can be seen that there is a region where the muon flux is suppressed: this region marked as W1 on Fig. 5f spans radially from the center toward the left bottom quadrant dividing the anomaly into two parts one identified by $x_1$ and $x_2$, and the other one with $x_3$ and $x_4$, due to the shadow effect of the load-bearing wall of the building located just above VP2.

## Discussion

The comparison between the measured and expected muon flux shown in Figs. 5 and 7 provides the evidence for a new empty structure and allows to estimate its size and position.

In Fig. 6 we report the profile plots for data (blue) and simulation (black) in order to better investigate the anomaly shape and its statistical significance. The highlighted regions correspond to the anomaly span indicated also by solid pink lines on the 2d plots. The error bars shows the statistical uncertainty derived from $1/\sqrt{n}$ where *n* is the number of muons per bin in the plots of Fig. 5. Since the statistics in the simulation was one order of magnitude higher than in the data, black line errors are negligible. In the anomaly region highlighted on the profile plots, the excess is clear on all profiles and cannot be explained by the statistical uncertainty. Thanks to the high statistical significance of each bin we can observe clearly the shape of the structures on the 2d distributions. In the other regions, a very good agreement is in general visible which supports a good description of the existing structures. The observed deviations of data plots from simulated ones reflect the difference in terms of shape and/or material density between the real site structure and the 3D-model used for the simulation. The excess in the simulation is clearly attributed to the shadow effect of the building walls missing in the model. There is another excess region in the data possibly due another unknown cavity which is less evident and revealed only in one projection, not sufficient for a 3D position estimation.

The anomaly location is consistent with the existence of a burial chamber, denoted as number 3 in Fig. 1, likely to be partially filled with alluvial material. The size of this empty region is expected to be $2\div 3.5$ m. The anomaly is clearly visible and its location is well-defined. The projection to the muography space tends to distort rectangular shapes, expected for a burial chamber, into quadrilateral shapes. In fact, we see a quadrilateral shape, thus indicating a structure of anthropogenic origin, consistent with a new burial chamber.

From the comparison of data with the expectations in all the other angular regions in Fig. 5, one can observe in general a very good agreement, with all the known structures detected. Nevertheless, there are regions where differences between data and simulation are visible. These differences can be attributed to the accuracy of the simulation model, due to the following reasons:

- No structural model of the nearby buildings, including their load-bearing walls, basements or other massive elements is available.
- The empty space is described with a null density while the rock with the constant value of 2.0 g/cm$^3$. This is a rough approximation as some underground regions are dominated by volcanic tuff, others by diluvial material while buildings basements are made of concrete. The actual density of these materials ranges between 1.4 and 2.4 g/cm$^3$.
- Not all empty underground spaces are described in our model. Underground connections, sewerage tombs and pipes are missing. It is also possible that some cellars of nearby buildings are missing from the model even though they lie within the acceptance region of the detectors.

These effects produce the small discrepancies between the expected and measured muon rates observed in some angular regions. Nevertheless, the size of these discrepancies cannot explain the observed anomaly. Firstly, the building walls create a sizeable effect when placed just above the detector since the muons travel a path as long as several meters inside the wall material. In this case the muon flux is strongly suppressed and a “shadow” is observed along this direction. This is the reason why a linear structure crossing the anomaly is visible in the plots (e, f) of Fig. 5. This effect is expected to decrease with the angle, and indeed it becomes negligible above 10 degrees. Secondly, soil density uncertainties result in a slight deviation of the rate in some angular regions but cannot produce a room-shaped anomaly. The cavity at the depth of 10 meters could not be confused with a cellar missing in the model, as they are typically located at the depth of 2–4 meters. Finally, the effect of missing pipelines or underground connections could result in a thin linear structure, incompatible with the observed anomaly. In fact we observe this kind of structure developing linearly toward the outer angular regions, marked as USP (Under Surface Pipeline) on Fig. 5c,f.

The reconstruction accuracy of the observed cavity could in principle improve by taking images from different angles, thus producing a tomography of the site. Nevertheless, the difficulty lies in the accessible sites for detector installations which are extremely limited in situ. The imaging of the site reported in this paper can thus be considered an optimal one.

## Methods

### Emulsion data reconstruction

The emulsion films used for this experiment were produced at Nagoya University. Each films is $25 \times 30$ cm$^2$ and consists of two sensitive layers, 70 $\mu$m thick each, deposited on both sides of a 175 $\mu m$ thick polystyrene base ^11^. AgBr crystals have a diameter of about 200 nm and the expected grain density for minimum ionizing particles tracks is on average about 40 grains per 100 $\mu$m path. After the emulsion development the grains size is of 0.6 $\mu$m. The readout of emulsion detectors requires high performance Scanning Systems ^12,13^ (SS), fully automated microscopes equipped with digital cameras and image processing chains. The whole thickness of the sensitive layers is spanned by adjusting the focal plane of the objective lens and a sequence of several tens of tomographic images is taken for each field of view at equally spaced depth levels. Emulsion images are then digitized, converted into a grey scale of 256 levels, sent to a vision processor board and analyzed to recognize sequences of aligned grains, i.e. clusters of dark pixels of given shape and size. Some of these spots are track grains; others, in fact the majority, are grains not associated to muon tracks, but rather to Compton electrons or produced by thermal excitation. The three-dimensional structure of a track in an emulsion layer (microtrack) is reconstructed by combining clusters belonging to images at different levels and searching for geometrical alignments. Each microtrack pair is finally connected across the plastic base to form the so-called base track. The typical resolutions for this type of emulsions in the detection of high energy (>1 GeV) charged tracks is of about 1 $\mu$m in position, 1.5 mrad in the $\phi$ angle and from 1.5 to 6 mrad depending on $\theta$ for the radial angle component  ^14^. So even a single 300 $\mu$m thick emulsion plate provides remarkably high angular resolution sufficient for most of muography applications. A detector module typically consists of at least two consecutive plates for the purpose of separating particle tracks integrated during exposure runs from other tracks. Reconstruction of a long track crossing several plates normally leads to an improvement in angular resolution. The next step is a pattern recognition procedure where the alignment of consecutive films with micrometric accuracy is achieved ^15^. The reconstruction efficiency depends on the emulsion thickness, grain density and the exposure conditions and in this experiment was higher than 94%. A small dependency of the efficiency on the $\theta$ angle is corrected in the post-processing phase. Two independent reconstruction and analysis chains were applied to different emulsion doublets. The SS used for *AnalysisI* ^16–19^ are different from those employed in *AnalysisJ* ^20,21^ in all respects, from hardware to software tools and track reconstruction algorithms. However, the final detection efficiency is similar and the angular distribution of muon tracks are fully compatible.

#### Angular acceptance for the emulsion detectors

Emulsion films register all charged particles passing through it, at any direction. As a result of the geometrical acceptance, the particle flux of muons crossing any flat surface decrease with the $\theta$ angle as $F_0 \cos \theta$, where $F_0$ is the flux orthogonal to the surface ($\theta =0$). Since $\theta$ regions close to $\pi /2$ (parallel to plate) show low significance due to the geometrical acceptance, upper limits on the $\theta$ range are normally applied in tracking algorithms, to reduce the processing time which is typically proportional to the solid angle, $2 \pi (1-\cos {\theta })$. This limit is set at $\tan \theta \le 0.8$ (round cut) for *AnalysisI* and $(\tan \theta _x \le 1.2) \& (\tan \theta _x \le 1.2)$ (square cut) for *AnalysisJ*. The two analyses differ also in the bin size of the angular histograms. In *AnalysisI*, a bi-dimensional squared bin with 0.01 bin size in the tangent space is used followed by a smoothing of the resulting histograms. In the *AnalysisJ*, the bin size is 0.025 without smoothing. The smoothing procedure is equivalent to a low-pass filtering with a $3\times 3$ core as used in image or signal processing to emphasise weak and wide signals: this first allowed to detect the cavity. On the other hand, the approach without smoothing does not introduce any distortion: sharp details such as wall shadows or room angles retain their original shape. Bin size selection depends also on the statistics. For the bin size of 0.01, significantly larger than the angular resolution quoted above, the statistics integrated in each bin is still sufficient: bins contained between 100 and 500 entries, in the peripheral ($\tan \theta = 0.8$) and in the central ($\tan \theta = 0)$) regions, respectively. The total accumulated statistics for each detector is 5 and 7 millions of tracks in the *AnalysisI* and in the *AnalysisJ* correspondingly in 28 days of exposure.

#### Coordinate system for data presentation

There are several ways, i.e. coordinate systems, used for the presentation of muography data. In this paper, we have adopted the projected angular slopes of reconstructed muon tracks, $\tan \theta _x$ versus $\tan \theta _y$, since they provide an intuitive picture, similar to the perspective projection used in conventional photographs. In doing that, the topology of the structures is approximately conserved and the comparison with an orthogonal site map becomes straightforward. Nevertheless, the binning inequality leads to a statistical suppression in the large $\theta$ regions due to the solid angle effect proportional to $1/cos^2\theta$. This may makes the interpretation of results more difficult in those regions without normalization. This is why we report the flux ratio, observed over expected.

#### Cavity position determination and reference points

The method used relies on the assumption that the trapezoidal contour of the observed structure in the angular space as reported in Fig. 5 keeps the same topological shape when seen from the two detector sites, except for some shift, rotation and deformation. Therefore, one can associate in the angular space the coordinates of the four corners observed from the two detectors: e.g. $x_1$ denotes the same corner in both detector views. For each corner, we have drawn in the 3D space two lines, one for each detector, originated from the detector position with the slopes defined by the corners themselves. We have then computed the points belonging to the two lines associated with the distance of closest approach and then we have computed the average point between the two. The same procedure is carried out for all 4 corners. The resulting points are the ones denoted as $x_1$...$x_4$ in Fig. 5 and Fig. 7. The uncertainty estimated by the above procedure is of 0.5 m in XY and 1.2 m in Z coordinate.

#### Muons flux generation and plots normalization

Muons are charged particles mostly produced by the decay of pions copiously produced in the interaction of high-energy cosmic rays (mainly protons) with the nucleons contained in the upper layers of the Earth atmosphere. The flux at the sea level is approximately uniform and constant, with small time and coordinate dependent variations, of the order of 5%. These variations affects mainly the low-momentum part of the spectrum, below 1 GeV, which does not impact our measurements because they do not reach our detector anyway.

There are several models describing the atmospheric muon flux and their spectrum. In the *AnalysisJ* the Miyake ^22^ parametrization was used, for the *AnalysisI* the Bogdanova’s ^23^ one.

Particles were traced through the 3D model using the Geant4 package^24^, from the detector position towards the sky. This approach of inverse propagation simplified and sped up the simulation. Two cases were simulated: a flat monolithic rock layer of 18 m depth above of the detectors without any building and any cavity; the complete 3D underground model with all known cavities and buildings. The inner structure of buildings was not available, so they were approximated by solid blocks with an average density equal to 6% of the concrete one. The muon flux expected with all building implemented as well as the observed data were normalized to the flux one would get with the bulky rock model. This ratio is shown as a function of the projected angular slopes in Fig. 5, where the relative excess in the flux caused by underground cavities and foreground buildings is clearly seen. This plot could be interpreted as a kind of picture of the internal structure where empty volumes appears in a yellow-red color while dense volumes in a green–blue one. The adopted normalization corrects binning-related $\theta$ dependencies and improves the sensitivity to deviations of the muon flux from its mean value.

## Data availability 

The datasets used and/or analyzed during the current study available from the corresponding author on reasonable request.
